# Factors Involved in Pain Perception and Quality of Life in Patients with Tropical Spastic Paraparesis

**DOI:** 10.1590/0037-8682-0291-2021

**Published:** 2022-06-06

**Authors:** Sebastian Criado-Martinez, Oriana Rivera-Lozada

**Affiliations:** 1 Universidad Privada San Juan Bautista, Professional School of Human Medicine, Lima, Peru.; 2 Universidad Norbert Wiener, South American Center for Education and Research in Public Health, Lima, Peru.

**Dear Editor:**

We have recently read an article published by Carvalho et al., in volume 52 of the present journal, in which they showed the effects of an exercise program on pain perception and quality of life in patients with tropical spastic paraparesis. The three study groups showed slightly improved pain perception even though one did not receive intervention and two groups showed reduced functional loss in the program^1^. Based on this, we observed the methodology. The recruitment of the participants complied with previously validated diagnostic criteria, and the exclusion criteria adequately delimited the population; however, the psychiatric impact on the study variables was not taken into account.

Depression is presented as a disorder of emotional and somatic scope, where up to 75% of patients feel pain. Chronic pain and quality of life are personal perceptions; hence, they can be influenced. Some factors previously identified are hope, acceptance of pain, and optimism, which have also been observed to affect functionality. However, it has been identified that there is bi-directionality in the interaction between depression and pain, with an increase in its perception in those who are depressed; this would be even more pronounced in patients with a prolonged early-onset disease, which is not rare^2^ ^,^ ^3^. 

Tropical spastic paraparesis is a chronic disease in which pain is frequent, and it has been previously demonstrated that patients suffering from it generally have depressive and anxiety symptoms associated with chronic pain^4^. Therefore, the participant's psychiatric condition would influence the measurement of the questions’ variables. Thus, other researchers such as Da Fonseca et al. excluded patients with psychiatric and neurological conditions when they tested an exercise program with the aid of virtual reality in the motor and cognitive improvement of patients with the same disease^5^.

In conclusion, participation in the psychiatric field should be considered when studying chronic diseases that generate pain and alter the quality of life since they predispose the research to generate inaccurate data due to the alteration in perception.
